# Contained hepatic vascular injuries following liver trauma: a retrospective monocentric study and review of the literature

**DOI:** 10.1097/JS9.0000000000001827

**Published:** 2024-07-04

**Authors:** Sébastien Frey, Imad Bentellis, Jacques Sedat, Florent Poirier, Patrick Baque, Damien Massalou

**Affiliations:** a Department of Emergency Surgery, Hospital of Pasteur 2, University Hospital of Nice; b University of Cote d’Azur; c Department of Urology, Hospital Pasteur 2, University Hospital of Nice; d Department of Interventional Radiology, Hospital Pasteur 2, University Hospital of Nice; e Department of Diagnostic Radiology, Hospital Pasteur 2, University Hospital of Nice, Nice; f Applied Biomechanical Laboratory, UMRT24, Université Gustave Eiffel, Aix-Marseille University, Marseille, France

**Keywords:** arteriovenous shunts, false aneurysm, hepatic vascular lesion, liver surgery, liver trauma

## Abstract

**Background:**

Over the past 30 years, there has been a major shift in the management of liver trauma. Contained hepatic vascular injuries (CHVI), including pseudoaneurysms and arteriovenous fistulas, are often feared because of the risk of secondary hemorrhage. However, little is known about CHVI. There are no guidelines for their management. Our aim was to validate the risk factors for CHVI, to identify the associated morbidities, and to establish a management protocol.

**Materials and methods:**

A retrospective study of 318 liver trauma cases from a level 1 trauma center over the past 15 years, comparing the presence or absence of CHVI. Univariable and multivariable analyses were conducted. The treatment used to manage CHVI was also compared.

**Results:**

Liver trauma with the following characteristics, AAST grade ≥III, bilateral injuries, and laceration-type lesions, were associated with a higher risk of CHVI. Grade AAST ≥III and bilateral injuries were confirmed in a multivariable study with odds ratios as high as 4.0 and 3.5, respectively. CHVI was associated with significantly more delayed bleeding and controlled computed tomography. After analyzing the noninterventional management of CHVI less than 2 cm, a management algorithm is proposed.

**Conclusions:**

This retrospective unicentric study and literature review provide additional insight into the patient profile at risk for developing CHVI, its associated morbidity, and its management.

## Graphical abstract

**Figure:**
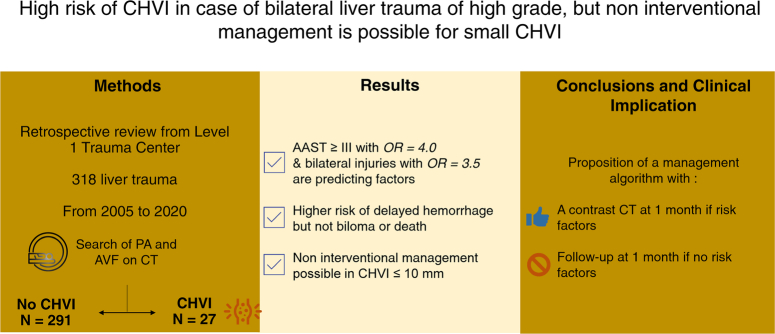

## Introduction

Highlights

- Limited hepatic vascular injury following liver trauma has an incidence of 8.5%.
- Two major risk factors were statistically validated: AAST grade ≥III (OR 4.02) and synchronous left and right hepatic injury (OR 3.53).
- Noninterventional management of vascular injury ≤10 mm is feasible and safe regardless of AAST grade.

Liver and spleen trauma are the two most commonly injured organs in abdominal trauma. The incidence of liver trauma varies worldwide, ranging from 2.95/100 000 population in developed countries to 13.9/100 000 population in developing countries^1,2^. In national databases, the incidence varies from 4.2 to 8%^3,4^. Hepatic trauma may result from either penetrating or blunt trauma. In both cases, the pathological lesions are classified into three cases: (i) intrahepatic vascular lesions, (ii) extrahepatic lesions, and (iii) bile duct lesions. Hepatic lesions are further described according to their types, their extension, and the presence or absence of major vascular injury^5^. Liver trauma can be fatal due to its fragile parenchyma and susceptibility to hemorrhage. Previously managed surgically with hepatic artery ligation or hepatic resection, it is now based on nonoperative management (NOM) with or without angioembolization (AE). This shift has been made possible by improvements in diagnostic imaging, interventional procedures such as angiography and embolization, and the use of damage control guidelines, resulting in a significant decrease in mortality^6,7^.

Contrast-enhanced computed tomography (CT) has become the cornerstone in the detection of hepatic vascular injury (HVI), including post-traumatic active hemorrhage and contained hepatic vascular injuries (CHVI)^8^. CHVI is represented by hepatic pseudoaneurysms (HPA) and arteriovenous shunts. HPAs occur when an injured artery leaks into the surrounding tissue, resulting in a contained hematoma, also known as a pseudoaneurysm. Arteriovenous fistulas (AVF) are rare and correspond to a communication between an artery and a vein. They may also be secondary to rupture of an HPA into the portal vein. In both cases, they are usually asymptomatic. However, they pose a significant risk of sudden major bleeding due to rupture. Clinical data on CHVI are scarce due to its rarity. To date, the risk profile for developing CHVI has not been described. Recommendations for the detection and treatment of HPA and AVF have not been established.

The aim of this study was to identify risk factors for CHVI and to investigate the management of CHVI after liver trauma.

## Materials and methods

### Study design

The study was reported according to the STROCSS criteria, Supplemental Digital Content 2, http://links.lww.com/JS9/C980 ^9^. This study was conducted in a verified level 1 trauma center. All abdominal traumas since 2005 are prospectively registered in a database. Trauma patients with liver injury between January 2005 and December 2020 were identified from this trauma registry and all medical records were carefully reviewed.

### Patient selection

Inclusion criteria were the presence of liver trauma, age greater than 15 years, and full-contrast abdominal CT. Liver trauma was identified by post-injury imaging or operative exploration. Admission after trauma could be either direct from the trauma site or by transfer from another regional hospital. On admission, if the patient was stable or stabilized after intensive care, a contrast-enhanced whole-body CT scan was performed. NOM was the primary treatment when possible. If the patient was unstable, primary surgical management was required. Surgical management could include single exploration, hemostatic techniques, hepatic packing, drainage, or hepatectomy. If we performed a damage control laparotomy with perihepatic packing, a second surgery was performed for definitive management after 24-48 hours. In such cases, a whole-body CT scan with contrast was performed after surgery. If a CT scan was not performed within the first 24 hours, the patient was not included in this study.

### Data collection

Clinical data, laboratory results, CT scans, and operative reports were extracted and analyzed retrospectively. Data analyzed included demographics, characteristics of liver trauma and its management. Demographic data included age, gender, mechanism of injury (penetrating or blunt), vital signs on arrival, mean Glasgow score on arrival, biological data on arrival, injury severity score (ISS), and associated visceral injury^9^. An ISS score greater than 15 was considered major trauma. Liver trauma was defined by its severity using the American Association for the Surgery of Trauma (AAST) liver injury grade (2018 revision)^10^, the type of injury, the number of segments involved, and the presence of active bleeding. Active bleeding was defined by contrast extravasation during the arterial or the portal phase of the CT scan, distinguishing an arterial bleed from a venous one, respectively. Injuries graded as AAST ≥III were considered severe. Treatment of liver trauma included the use of radiologic embolization, nonoperative treatment, and/or surgical treatment. Follow-up data included need for blood transfusion, intensive care unit (ICU) days, total hospital stay, incidence of acute liver failure (ALF), bile leakage (BL), secondary bleeding, and death. Morbidity was categorized according to the Clavien–Dindo grading system^11^. ALF was defined by the presence of acute encephalopathy and an INR ≥1.5. BL was defined as the presence of discharge through an intra-abdominal fluid drain (bilirubin concentration ≥3x the serum bilirubin concentration), the presence of fever and/or abdominal pain associated with intra-abdominal fluid accumulation detected by CT, or cases requiring radiologic intervention or laparotomy due to bile collection or biliary peritonitis^12^. Secondary hemorrhage, or delayed hemorrhage, was defined as bleeding from liver trauma more than 24 hours after the time of injury with (i) a drop in hemoglobin >3 g/dL and associated increase in volume of subcapsular or intraparenchymal hematoma or hemoperitoneum [with Hounsfield unit (HU) density consistent with blood: 30-80 HU] on repeat CT compared to the initial scan; (ii) using the AAST radiologic definition of active bleeding with vascular contrast extravasation, focal or diffuse, that increases in size or attenuation on delayed phase; (iii) intraoperative findings of active or recent (fresh blood) hemorrhage from liver injury in a patient with hemodynamic instability brought directly to the operating room without preoperative imaging. Laboratory monitoring, including hemoglobin, was performed daily during ICU stay and every other day during inpatient admission to the hospital floor.

### Outcome measures

The primary outcome of interest was the identification of CHVI on either the initial or follow-up CT scan. Follow-up contrast-enhanced CT was performed on day 5 for NOM. If NOM included AE, follow-up CT was scheduled 5 days after AE to verify the absence of focal collection of vascular contrast. For grade ≥IV injuries, an additional abdominal CT could be performed on day 10 after admission or AE. CHVI was defined as HPA and/or AVF, according to the AAST organ injury scale (2018 revision) for liver trauma definition: a contained focal collection of vascular contrast that decreases in attenuation on delayed-phase images on contrast-enhanced CT^10^. A junior and a senior radiologist, blinded to the diagnosis, reviewed all initial and follow-up CT scans.

### Statistical analysis

Statistical analyses were performed using R software (R Foundation, Vienna, Austria)^13^. Quantitative variables are presented as mean ± standard deviation or median and interquartile range for small sample sizes (<30). Student’s t-test or Wilcoxon signed rank test were used depending on the sample size. Qualitative variables are presented as numbers and percentages. Chi-squared test, Fisher’s exact probability test or Kruskall-Wallis test were used. Logistic regression was performed to explore the potential risk factors for CHVI among AAST grade, type of injury, side of injury, number of segments injured, active bleeding, and ISS score. All analyses were bilateral, and the alpha risk was set at 0.05.

### Ethical statements

The study was performed in compliance with the STROBE and STROCSS guidelines (Supplemental Digital Content 1, http://links.lww.com/JS9/C979). The study was approved by the local Ethics Committee (Ethical Committee N°112) and complied with the 1964 Helsinki Declaration and its later amendments^14^. Informed consent was not required because of the retrospective nature of the study and the minimal risk involved. The study protocol was published on a research registry.

## Results

**Figure 1:**
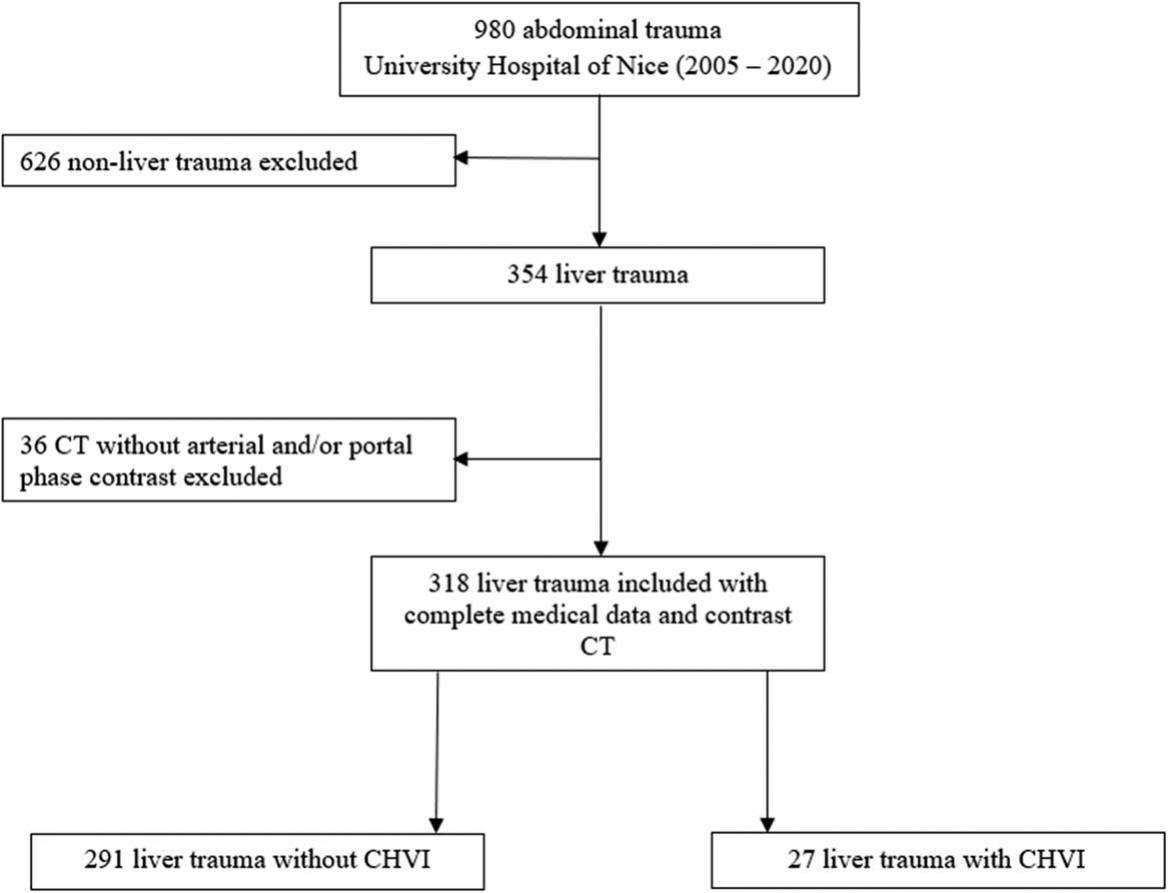
Flowchart.

From January 2005 to December 2020, 980 abdominal trauma patients were admitted to our hospital. Three hundred and fifty-four patients had liver trauma and 318 of them fulfilled the inclusion criteria (Fig. 1). The cohort was predominantly male (71.1%) with a mean age of 35 years. Nonpenetrating trauma was the most common type of trauma (84.3%). The three main etiologies were motor vehicle collision in 63.5%, fall in 17.9%, and knife injury in 9.2%. Nineteen patients (6.0%) were referred from other institutions. On admission, 30.8% had hemodynamic instability.

**Figure 2:**
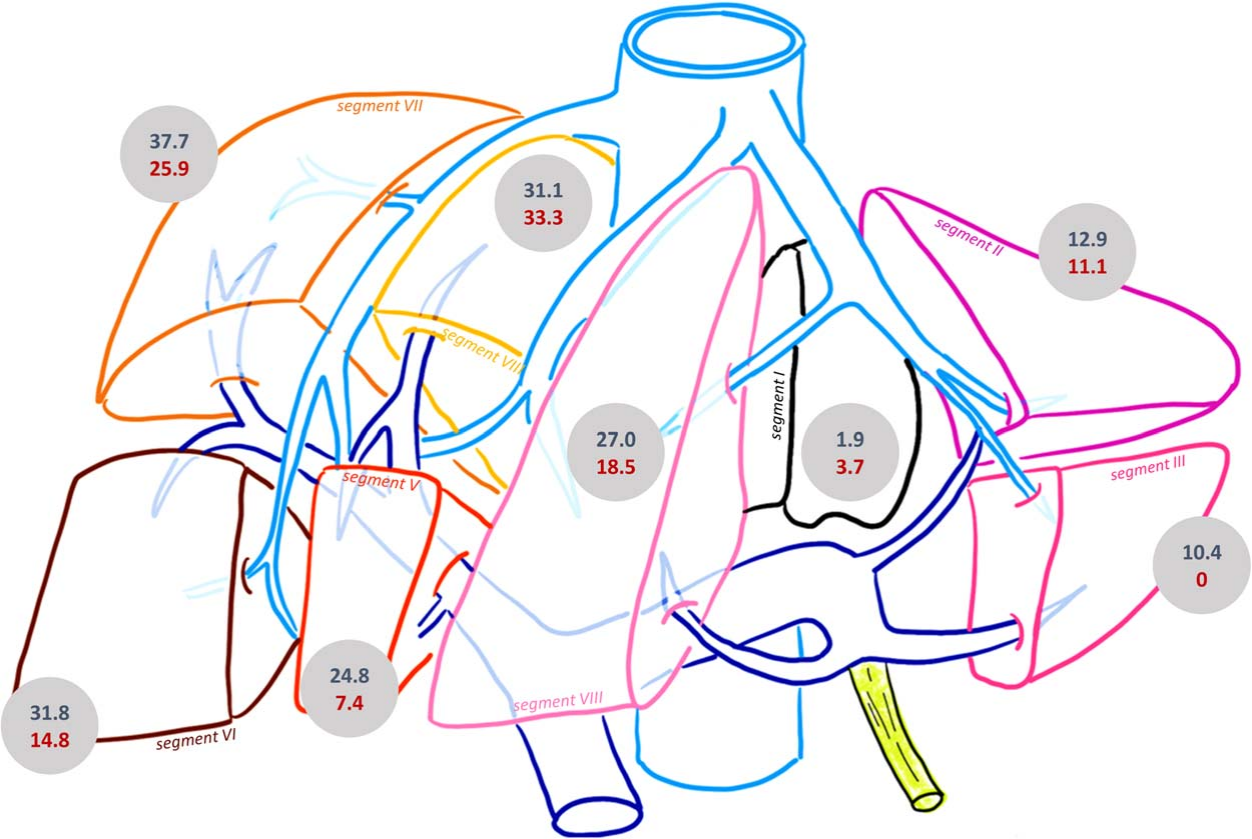
Distribution of the percentage of liver trauma (in blue) and CHVI (in red) per segment. CHVI, Contained hepatic vascular injuries.

Twenty-seven (8.5%) patients were diagnosed with CHVI, 20 with HPA (74.1%), and eight with AVF (25%), with one patient having both. Five HPA (25%) and two AVF (25%) were diagnosed on the initial CT scan. The mean time to diagnosis was 6.5 days (0–31 days) after admission. The mean size was 12.6 mm (3–46). The percentage of CHVI in liver trauma per AAST grade increased with the severity of liver trauma. The higher the AAST grade, the more CHVI occurred. The distribution of liver trauma and CHVI per liver segment is also shown, highlighting segments VI, VII, and VIII as the main localization of injury (Fig. 2).

The 27 patients with CHVI were compared with the remaining 291 patients without CHVI. Overall, demographics and trauma characteristics were comparable between the two groups, with the exception of mean ISS (Table 1). The mean ISS was 27, and in both groups, it was greater than 15, which is the threshold for severely injured patients.

**Table 1:** Demographic data and trauma features.

	CHVI − n=291	CHVI + n=27	*P*
Age, mean (years)	35.4	34.6	0.795
Male sex, *n* (%)	205 (70.4)	21 (77.8)	0.561
Mechanism of injury, *n* (%)	–	–	0.134
* *Motorbike accident	117 (40.2)	9 (33.3)	–
Car accident	30 (10.3)	2 (7.4)	–
Walking accident	14 (4.8)	1 (3.7)	–
Other transportation accident	25 (8.6)	3 (11.1)	–
Stabbing wound	25 (8.6)	4 (14.8)	–
GSW	10 (3.4)	4 (14.8)	–
Fall	55 (18.9)	2 (7.4)	–
Other	15 (5.2)	2 (7.4)	–
Penetrating trauma, *n* (%)	42 (14.4)	8 (29.6)	0.072
Hemodynamic instability upon arrival, *n* (%)	93 (33.6)	5 (19.2)	0.202
Mean Glasgow score, mean	13	14	0.060
Biological data upon arrival, mean	–	–	–
Hb	11.7	12.8	0.063
pH	7.28	7.34	0.053
Arterial lactate	3.74	2.59	0.150
ISS	**27.2**	**19.2**	**0.015**
Associated visceral injury, *n* (%)	–	–	–
Spleen	67 (23.0)	1 (3.7)	0.309
Kidney	74 (25.2)	5 (18.5)	0.748
Thoracic cage or lungs	178 (61.2)	14 (51.9)	0.459
Vertebral columnar	60 (20.7)	2 (7.4)	0.158
Bones	115 (39.8)	6 (22.2)	0.112
Pelvis	56 (19.3)	3 (11.1)	0.430
Brain or spinal cord	54 (18.6)	2 (7.4)	0.231

Bold values are statistical significance *P*<0.05.

GSW, Gunshot wound; Hb, Hemoglobin; ISS, Injury severity score.

### Identification of risk factors

**Figure 3:**
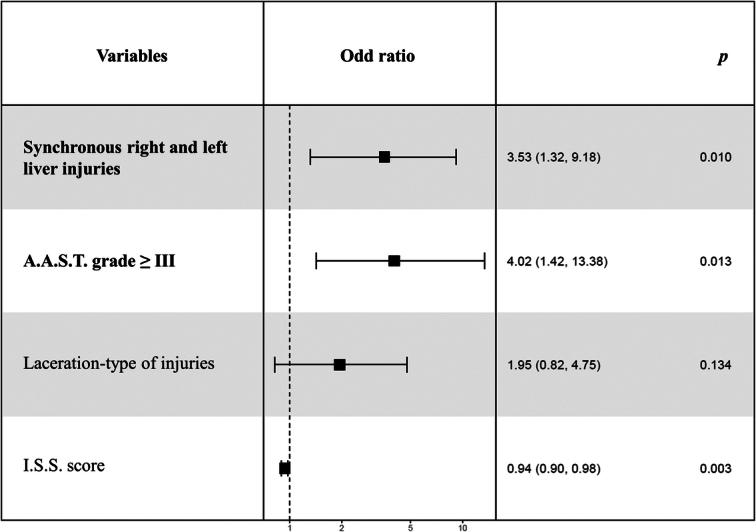
Multivariable analysis of the main risk factors of CHVI.

CHVI was associated with AAST grade ≥III (81.5 vs. 52.9, *P*=0.008), type of injury (59.3 vs. 33.2%, *P*=0.013), and bilateral liver injury (left and right liver) (33.3 vs. 13.6%, *P*=0.015). Laceration was the most common type of injury in CHVI. The number of liver segments injured was not statistically different between the two groups. Active bleeding on the initial CT scan was not associated with CHVI (12.7 vs. 18.5%, *P*=0.319) (Table 2). Multivariable analysis confirmed the association between CHVI and AAST grade ≥III and bilateral liver injury, with odds ratios of 4.02 (95% CI: 1.42–13.38, *P*=0.13) and 3.53 (95% CI: 1.32–9.18, *P*=0.10), respectively (Fig. 3). However, the laceration type of injury was not significantly associated (OR=1.95, 95% CI: 0.82–4.75, *P*=0.13). The ISS score had a positive association with an odds ratio of 0.94 (95% CI: 0.90–0.98, *P*=0.003).

**Table 2:** Liver trauma characteristics and management.

	CHVI − n=291	CHVI + n=27	*P*
Characteristics of liver trauma upon admission			
AAST grade, *n* (%)	–	–	–
I–II	137 (47.1)	5 (18.5)	
III–V	**154 (52.9)**	**22 (81.5)**	**0.008**
Type, *n* (%)			
Hematoma	189 (65.2)	11 (42.3)	
Laceration	**94 (33.2)**	**16 (59.3)**	**0.013**
Mixt	4 (1.4)	0 (0.0)	–
Tearing	2 (0.7)	0 (0.0)	–
Side of the liver concerned			**0.019**
Right	161 (59.0)	14 (51.9)	–
Left	75 (27.5)	4 (14.8)	–
Both	**37 (13.6)**	**9 (33.3)**	**0.015**
Number of segments, *n* (%)			
1	60 (20.6)	1 (3.9)	
>1	156 (56.9)	19 (70.4)	0.252
>2	49 (17.9)	5 (18.5)	1.000
>3	26 (9.4)	2 (7.4)	1.000
Active bleeding, *n* (%)	37 (12.7)	5 (18.5)	0.319
Arterial	24 (8.2)	2 (7.4)	–
Venous	13 (4.5)	3 (11.1)	–
Follow-up and post-traumatic morbimortality			
RBC transfusion, *n* (%)	116 (45.0)	8 (30.8)	0.237
FFP transfusion, *n* (%)	87 (29.9)	4 (14.8)	0.261
ICU admission, n (%)	**236 (81.4)**	**17 (63.0)**	**0.042**
Length of stay in ICU, days	8	7	0.648
Total length of stay, days	16.6	17.8	0.740
Clavien–Dindo	–	–	**0.004**
I–II	28 (9.7)	8 (30.8)	
III–IV	72 (25.0)	8 (30.7)	
Bilioma, *n* (%)	14 (4.8)	4 (14.8)	0.086
Liver insufficiency, *n* (%)	1 (0.3)	0 (0.0)	1.000
Delayed hemorrhage, *n* (%)	**4 (1.4)**	**5 (18.5)**	**<0.001**
Death, *n* (%)	37 (12.8)	1 (3.8)	

Bold values are statistical significance *P*<0.05.

AAST, American association for the surgery of trauma; FFP, Fresh frozen plasma; RBC, Red blood cells.

### Outcomes analysis

Management was similar in both groups with regard to NOM (58.8 vs. 58.8%, *P*=1.0) or surgical treatment (Table 2). NOM for liver trauma was successfully performed in 77.7% of the CHVI-group and 64.3% of the CHVI+group. AE was performed in 70 (22.0%) cases overall, on day 3 of admission on average. For 15 patients, AE came in completion to the surgical treatment due to active and persistant arterial bleeding. Indications for AE were active vascular bleeding (77.1%), CHVI (18.6%), and delayed hemorrhage (4.3%). AE was performed more often in the CHVI group (44.4 vs. 20.3%, *P*=0.027). Surgical liver management was required for 68 (21.4%) patients overall. Specific management included hepatic packing (31.6%), selective hemostasis (12.0%), liver resection (0.8%), and surgical drainage (6.8%). The different techniques used did not differ between the two groups (*P*=0.761).

Postoperative follow-up and morbidity are summarized in Table 2. The distribution of Clavien–Dindo grades was different between the two groups, with more grade I and less grade V in CHVI. Biliary leak and hepatic insufficiency were similar, although biliary leak was more common in CHVI (14.8 vs. 4.8%). Secondary bleeding occurred in nine patients (2.8%), with five (18.5%) in the CHVI group (*P*<0.001). Two patients required damage control laparotomy, two had an AE (one having both surgery and AE), and six were managed conservatively. Mortality was similar in both groups. Only one death corresponded to a patient with a diagnosed AVF, but it occurred due to multiple organ failure.

### Management of CHVI

The type of management of CHVI was further compared (Table 3). We identified two types of management: the noninterventional management (NIM) group (*n*=13) and the interventional management (IM) group (*n*=14), including AE or surgery. In the NIM group, the size of the CHVI was less than or equal to 10 mm in 12 patients. The remaining patient had a size of 25 mm. Follow-up CT scans showed spontaneous thrombosis in all cases, 9 (69.2%) at 1 month and 4 (30.8%) at 2 months. There were three delayed hemorrhages, two of which occurred before the diagnosis of CHVI. The one occurring after the diagnosis of CHVI resolved following blood transfusion and did not require interventional management. Overall, no patient from the NIM group required an interventional procedure. In the IM group, the size of the CHVI was greater than 10 mm in all cases. Thirteen patients were treated with AE and one required surgery. The latter had a large HPA, diagnosed on the initial CT scan, that progressed to a rupture requiring urgent laparotomy. For the remaining patients, AE was effective in 12 patients (92.3%) who were checked on a systematic CT scan 5 days after AE. The patient with failed AE had a spontaneous thrombosis at the 2-month follow-up CT scan. Finally, two delayed hemorrhages occurred in this group, with one before the diagnosis of CHVI, and one upon the diagnosis of CHVI, motivating AE.

**Table 3:** Comparison of the type of management in the case of CHVI.

	NIM (*n*=13)	IM (*n*=14)	*P*
AAST grade, *n* (%)	–	–	–
I–II	2 (15.4)	3 (21.4)	1.000
III–V	11 (84.6)	11 (78.6)	1.000
Type, *n* (%)			
Hematoma	5 (38.5)	6 (42.9)	1.000
Laceration	8 (61.5)	8 (57.1)	1.000
Size of CHVI, mm	**8 [3–25]**	**18 [11–46]**	**<0.001**
Day of diagnosis following trauma, days	3.5	6.5	0.349
Length of stay in ICU, days	4	4.5	0.641
Total length of stay, days	9	15	0.119
Bilioma, *n* (%)	1 (7.7)	3 (21.4)	0.644
Delayed hemorrhage, *n* (%)	3 (23.1)	2 (14.3)	0.927

Bold values are statistical significance *P*<0.05.

IM, Interventional management; NIM, Noninterventional management.

## Discussion

Scientific data on CHVI in liver trauma are scarce (Table 4). Based on 318 liver injuries over the last 15 years, we found an incidence of CHVI of 8.5%, in line with the overall incidence of 1.2–11.3% (Table 4). This study represents the largest series of CHVI. Two major risk factors were identified and validated by multivariable analysis: the presence of AAST grade ≥III (OR=4.02) and the presence of simultaneous right and left liver injury (OR=3.53). Of note, the penetrating nature of the injuries was not identified as a risk factor. The association between the severity of liver trauma and the development of CHVI has been previously reported in the literature^16,18,22^. Although less frequent, vascular malformations in less severe trauma should not be neglected, as was the case in five of our patients. Nevertheless, our study confirmed the statistically significant association between AAST grade ≥III and CHVI. Without underestimating low-grade injuries, this relationship is probably related to the depth of the lesion within the liver parenchyma. CHVI were also significantly more frequent in cases of synchronous right and left liver involvement. Interestingly, this finding was not related to the number of segments involved. Nevertheless, hepatic trauma involving both the right and left livers are more likely to be central injuries, which have a denser vascular network and higher arterial pressure. It is also worth noting the possibility of diagnosing CHVI on initial imaging^15^. In our study, one in four patients was diagnosed. In the recent Auckland study, this may be as high as 43% of patients. This underscores the importance of performing an injected CT scan on admission or postoperatively in case of initial surgical management. On the other hand, several elements were not identified as risk factors. The mechanism of trauma (penetrating or nonpenetrating) and the type of liver injury (hematoma or laceration) were not identified as risk factors in the multivariable analysis. Although penetration and laceration have been identified as two variables favoring the development of CHVI, their statistical association has never been established^8^. A large multicenter cohort of CHVI may eventually confirm this trend.

**Table 4:** Systematic review of retrospective studies of CHVI from liver trauma.

First author	Year	Number of liver trauma	Number of CHVI	Incidence	Age – Sex	Type of injury	Type of CHVI	Interval between trauma – CHVI (d)	Size (mm)	Treatment	Survival
Our study	2023	318	27	8.5	34.6 – 21M/6F	19 NP 8 Penetrating	IHPA	6.5 (0–31)	12.3 (2.5–46)	13 NIM 13 AE 1 Surgery	1 death
Henry^15^	2022	450	7	1.6	–	–	PA	6 (0–16.9)	9.5 (2–17)	5 AE 2 Surgery	Yes
Kagoura^16^	2022	176	3 AAST I-II 7 AAST III-V	5.7	–	–	PA	–	4.5 AAST I–II 6.5 AAST III–V	4 NIM – 4 thromboses 6 AE	Yes
Lada^17^	2021	171	12	7.0			PA - AVF				1 death
Wagner^18^	2020	634	18 AAST III-V	2.8	–	12 NP 6 Penetrating	PA	6.5 (4–9)	–	4 NIM – 4 thromboses 14 AE 2 Surgery	2 death
Kittaka^19^	2015	62	7	11.3	6M/1F	–	PA	5 (3–7)	7.7 (2–11.9)	6 NIM – 6 thromboses 1 AE	Yes
Østerballe^20^	2014	259	7	2.7	30.4 – 5M/2F	7 NP	IHPA		–	7 AE	Yes
Forlee^21^	2004	–	8		26 – 7M/1F	1 NP 7 Penetrating	IHPA	15 (1-60)	–	1 NIM 7 AE	–
Croce^22^	1994	482	2 AAST I-II 4 AAST III-V	1.2	24 – 5M/1F	3 NP 3 Penetrating	IHPA	—	—	6 Surgery	2 death

AE, angioembolization; AVF, arteriovenous fistula; d, days; IHPA, Intrahepatic pseudoaneurysm; NIM, noninterventional management; NP, nonpenetrating; PA, pseudoaneurysm.

The morbidity and mortality associated with liver trauma are not negligible. It also directly correlates with AAST grade. First, the incidence of bile leak was not statistically different between the two groups, although there was a trend in the case of CHVI. This association was significant in the Auckland study with a *P*-value of 0.0045, although there were only three cases of biliary leakage among seven HPA^15^. A direct association between CHVI and biliary leakage may be difficult to establish for several reasons: (i) surgical management and severity of trauma are two factors favoring the occurrence of biliary leakage, ranging from 4 to 5% (ii) AE has a high risk of hepatic necrosis, which in turn may lead to biliary leakage^16^. In Oo *et al*.^23^, AE was statistically associated with bilioma with an OR of 16. Second, the relationship between CHVI and delayed hemorrhage has been accepted for many years in the case of trauma to solid organs (spleen or liver). However, it remains rare and difficult to assess a direct relationship between brutal hemorrhage and CHVI^24^. For example, in our study, three of the five cases of delayed hemorrhage in the CHVI group occurred after the diagnosis of CHVI. As Henry and Fichher^15^ called it, bleeding caused by CHVI is essentially insidious. In their analysis, CHVI had an increased risk of bleeding, with a drop in hemoglobin and a significantly higher transfusion rate at follow-up, but without an increased risk of secondary rupture and/or delayed acute bleeding. This clinical difference can be explained by the pathology of the hepatic vascular lesions: whereas extrahepatic HPA or AVF develop within a wall comprising each layer of a vascular wall, CHVI results from intraparenchymal arterial bleeding controlled by the surrounding liver tissue, defining the true ‘false aneurysm’. Regarding mortality, Wagner *et al*.^18^ reported an unfavorable outcome in 50% of cases of hemorrhagic shock. In contrast, only one death was reported in the last three published studies^15–17^. In our study, only one case of hemorrhagic shock directly attributable to HPA occurred, the other being due to another cause. The presence of CHVI did not confer an excess risk of mortality, even in cases of severe AAST grade. Overall, the pooled mortality across all retrospective studies is 5.9%, which justifies current management with interventional strategies and/or close monitoring (Table 4).

**Figure 4:**
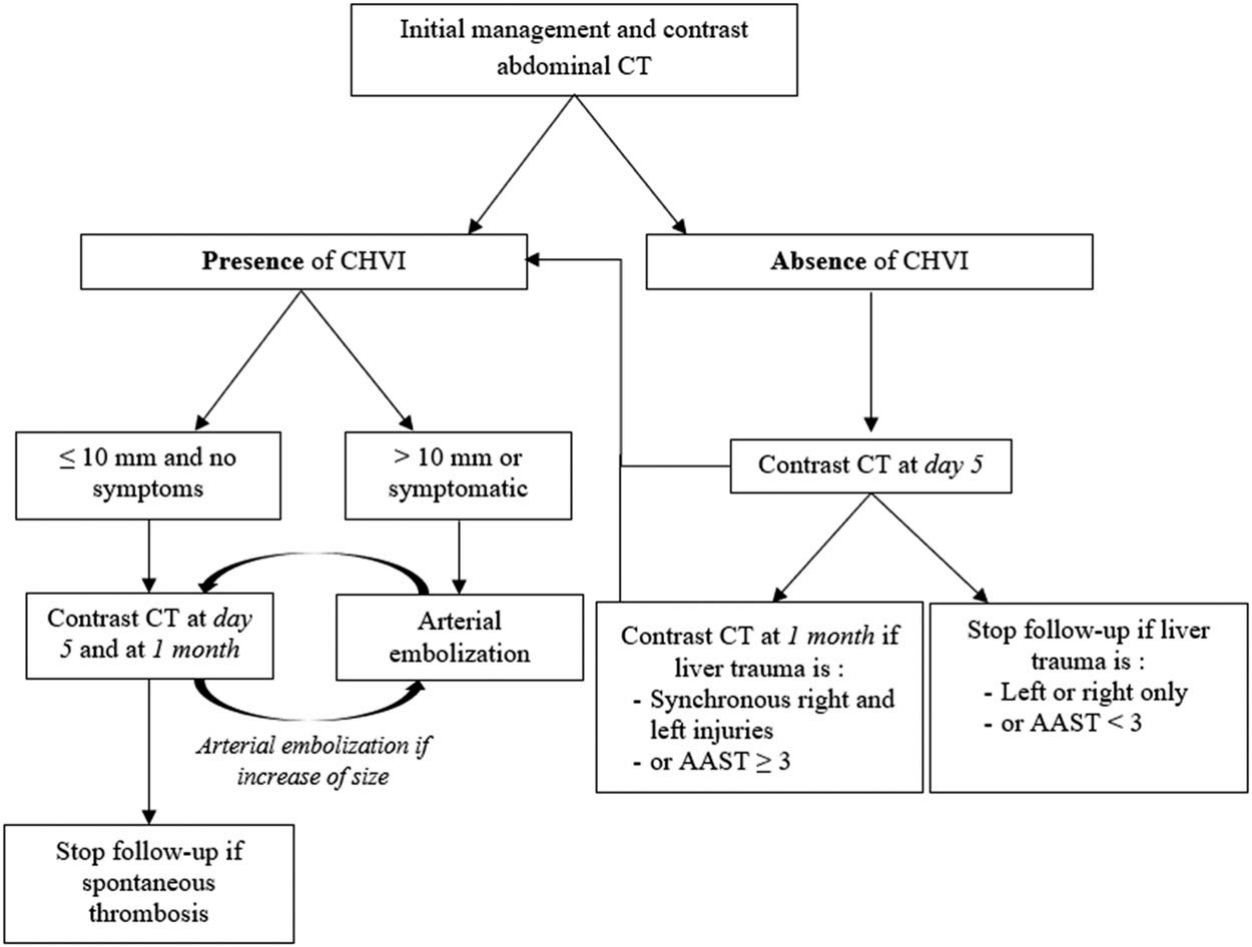
Algorithm for the management of CHVI CHVI, Contained hepatic vascular injuries.

The management of CHVI is essentially threefold: surveillance alone, AE, and surgical treatment. A review of the literature, including our study, shows that these strategies are used in 25.5, 60.0, and 14.5% of cases, respectively (Table 4). The use of AE in CHVI represents the current standard of care, essentially based on extrapolation from studies of true visceral artery aneurysms. In the absence of objective data, it is common practice to embolize symptomatic CHVI (signs of rupture) and/or larger than 10 mm and/or AAST grade ≥III^15,16^. Noninterventional treatment has gained attention in recent years. An original study from the Mayo Clinic demonstrated the feasibility of noninterventional treatment of splenic PA in cases of PA <20 mm, asymptomatic, and in patients other than women of childbearing age^25^. However, in the context of splenic trauma, it has also been demonstrated that spontaneous thrombosis of the PA occurs in one out of two cases^26^. More recently, Kittaka *et al*.^19^ demonstrated that in post-traumatic PA of the liver, spleen, and kidney, spontaneous thrombosis occurs in all cases when its diameter is less than 10 mm, at an average of 8 days. This was the only study that reported the rate of spontaneous thrombosis in liver trauma, but it included only six cases. Accordingly, our study adds further data with spontaneous thrombosis in all cases when smaller than 10 mm and in one case at 26 mm. These results were independent of AAST grade, as in included over 78.6% of AAST grade ≥III. Therefore, based on these results, we propose a decision algorithm to assist in the management of CHVI after liver trauma (Fig. 4).

Our study has several limitations. First, the retrospective, observational nature of the study means that we can only extrapolate our results with a level of evidence III. However, a prospective study in this context seems difficult given the rarity of the event studied. Despite the prospective data collection, only 27 events occurred in 15 years. Second, the rarity of the event also increases the statistical risk of a type II error, although the total number of CHVI is the largest to date in a total cohort of over 318 patients. Third, both penetrating and nonpenetrating trauma were included in this study. This choice was made to represent the day-to-day activities of general emergency departments. Finally, the single-center nature of this study represents an additional bias. A multicenter or national study could help to establish a ‘state of the art’ in the management of this pathology. However, it would be difficult to conduct a prospective study in this low incidence, emergency setting. Nevertheless, this study represents an important synthesis in establishing a risk profile, providing additional information on associated complications, and highlighting the possibilities of noninterventional treatment in the management of CHVI.

## Conclusion

Due to its low incidence, knowledge about CHVI after liver trauma is scarce and its management is not yet codified. Through a unicentric analysis and literature review, this study provides new data on risk factors, associated complications, and management options. CHVI occurred mostly in severe AAST grade, but was also possible in nonsevere stages. Its incidence was increased when liver injury occurred simultaneously on the left and right side. Penetrating nature and type of injury seemed to increase the risk of CHVI, but did not reach significance. The presence of these factors should prompt more careful radiologic screening in the surveillance of such patients. The need for such close monitoring is justified by the risk of secondary bleeding, which is responsible for a mortality rate of about 5%. Finally, AE is the main therapeutic modality, sometimes at the cost of hepatic necrosis and biliary leakage. Noninterventional management is possible for CHVI smaller than 10 mm, regardless of AAST grade, leading to spontaneous healing.

## Ethical approval

This study complied with the standards of the Declaration of Helsinki. Ethical approval for this study (Ethical Committee N°112) was provided by the local Ethical Committee of the University Hospitals, named ‘Département de Recherche Clinique et Innovations’.

## Consent

Not applicable.

## Source of funding

Not applicable.

## Author contribution

S.F.: conceptualization, investigation, data curation, methodology, formal analysis, and writing – original draft; I.B.: data curation and formal analysis; F.P.: investigation and data curation; J.S.: investigation and data curation; P.B.: writing – review and editing; D.M.: conceptualization, investigation, data curation, methodology, formal analysis, project administration, visualization, and writing – original draft. All authors read and approved the final manuscript.

## Conflicts of interest disclosure

The authors declare no conflicts of interest.

## Research registration unique identifying number (UIN)

NCT06249646.

## Guarantor

Sebastien Frey – Department of Emergency Surgery, Hospital Pasteur 2, University of Cote d’Azur, 30, Voie Romaine 06000 Nice, France. E-mail: freysebastien6@gmail.com.

## Data availability statement

Not applicable.

## Provenance and peer review

Not applicable.

## Supplementary Material

SUPPLEMENTARY MATERIAL
